# Modeling the Simultaneous Effects of Particle Size and Porosity in Simulating Geo-Materials

**DOI:** 10.3390/ma15041576

**Published:** 2022-02-20

**Authors:** Jichao Sun, Yuefei Huang

**Affiliations:** 1 School of Water Resource & Environment, China University of Geosciences, Beijing 100083, China; 2 State Key Laboratory of Hydroscience & Engineering, Tsinghua University, Beijing 100084, China; yuefeihuang@tsinghua.edu.cn

**Keywords:** joint particle, particle size and gradation, particle discrete element method, numerical simulation

## Abstract

The particle discrete element method (PDEM) is widely used to simulate rock and soil materials to obtain stress and strain. However, there are three shortcomings: (1) Single sphere or ellipsoids directly replace the soil particles; (2) it treats the diameters of spheres or ellipsoids as the soil particle size; (3) the overlapping particle volume is not deducted in calculating the porosity. Hence, it is difficult for the simulation of the geological body to agree with reality. This research found a rotation calculation model and a pixel counting method to make joint soil particles more accurately simulate geological materials to solve the three shortcomings. The model successfully obtained the gradation curve and porosity of the simulated geological body with joint particles. This research will further enrich and broaden the application prospects of PDEM and provide a reference for scientific research and engineering fields in geological engineering, geotechnical engineering, and petroleum engineering.

## 1. Introduction

In order to obtain the mechanical properties of rock and soil mass, a large number of in situ and laboratory tests are carried out during the construction of hydraulic and geotechnical engineering. The cost of many in situ and laboratory tests is high. After the on-site sampling, the indoor test cannot guarantee the sample’s original stress; the test results largely deviate from the actual one. Simultaneously, different lithologies and sampling locations make it impossible to apply these test results widely. The numerical calculation method can yield geomechanical parameters, including stress–strain and porosity at specific depths under these standard preconditions to achieve these goals: low costs, maintaining the original stress and simulating different soils and rock.

Some methods can model geological materials, mainly the finite element method (FEM) and the discrete element method (DEM). The finite element method has solved many theoretical, technical, and engineering problems in geology [1,2]. However, many issues still cannot be solved. First, the finite element method is unsuitable for large deformation [3]. Second, the discontinuous rock mass cannot use the technique, as these bodies contain voids, cracks, and fissures. Third, this method cannot directly obtain the rock and soil porosity. The following formula containing stress–strain parameters calculates the porosity. The strain and stress result from the finite element method of analyzing soils/rock. Then, the new porosity is Equation (1).

(1) $$n_{new}=\frac{n_{0}E-(1-2\upsilon )\Theta }{E-(1-2\upsilon )\Theta }$$

where $n_{new}=\frac{e_{0}-\Delta }{1+e_{0}-\Delta }$, $n_{0}=\frac{e_{0}}{1+e_{0}}$, $\theta_{t}=\frac{1-2\upsilon }{E}\Theta$, $\theta_{t}=\frac{\Delta }{1+e_{0}}$, *n*_0_ is the original porosity, *E* is the elastic modulus, *υ* is the Poisson’s ratio, *e*_0_ is the original void ratio, Δ is the pore change/reduction, *e*_0_ is the original void ratio, Θ = σ*_x_*+ σ*_y_*+ σ*_z_*, σ*_x_*, σ*_y_*, and σ*_z_* are the stress in the *x*, *y,* and *z* direction, *θ_t_* is the volumetric strain, and *n_new_* is the new porosity. The void is pressed and reduced, but the volume of the soil/rock particles does not diminish. So, the new porosity is calculated by the stress/strain. The method is not direct.

The discrete element method was proposed by Cundall and Strack [4] and was widely used in analyzing large rock and soil deformation. The method can solve significant deformation problems simulate a discontinuous body and granular soils [4]. Furthermore, soils contain many cracks, faults, micropores, and microchannels and are not continuous or uniform bodies. There are many mechanical interactions between cracks and particles [5,6]. The discrete element method is widely used in geotechnical, geological, and hydraulic fields [7,8].

**Figure 1:**
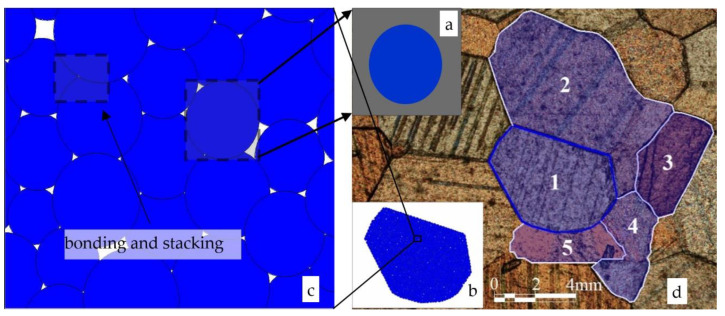
Polarized microscopic marble slice and crystal particle simulation (**a**) Single-particle/circular particle/ball particle; (**b**) one crystal that is a joint particle/crystal particle; (**c**) partial enlargement of a joint particle; (**d**) crystal in marble slice. 1, 2, 3, 4, and 5 are five crystals in a marble slice, this scale is approximate scale.

Specifically, the discrete element method has some branches. For example, the commercial software of the sphere discrete element method [9,10,11,12] is PFC and Yade, esys-particle and LIGGGHTS (Figure 1); the commercial software of the discontinuous medium (jointed rock mass) discrete element method [13,14,15] is UDEC/3DEC, FLAC and EDEM, the discrete lattice points discrete element method [2,16], such as Xsite. However, the particle discrete element method (PDEM) is more widely used in materials processing.

The research on the particle discrete element method (PDEM) [2,9,17] has achieved fruitful results, such as the reference parameters of the Brazilian disc failure test and PFC2d simulation [9], the laws of various mechanical parameters, the coupling relationship with fluids [12,18], the relationship between the particle size and equilibrium position for the particles in the fluid channel [19], the deformation characteristics of geological bodies, and disaster simulations [20], there are many mechanical interactions between cracks and the particles [5,6]. The method measures particle velocity in water and multiphase flow by particle image velocimetry [21,22]. These studies simulate the soil’s circular particles and apply boundary conditions to the circular soil to obtain physical and mechanical results under deformation and stress conditions.

The combination of multiple spherical particles can simulate natural soil particles. For example, Sun [12] successfully used different quantities and radii particles to simulate various gravel. Figure 1 shows the connection and composition simulation process from single/circular/ball particles to soil/rock. Many single particle/circular particle/ball particles (Figure 1a) made up a joint particle/crystal particle (Figure 1b). The connection method between single particles is bonded and stacked (Figure 1c). Moreover, many joint particles (Figure 1b) make up soils/rock (Figure 1d). The joint particles (Figure 1b) are polygonal and not circle/ball/sphere.

DEM uses particles to simulate soils. The simulation is successful when some parameters and results are consistent or the same. In soil mechanics, two crucial indicators of soil mass are particle size and particle gradation. Past research [23] does not concern the particle gradation, or it directly takes the circle diameters as the particle size [24], or the particle radius is adjusted slightly with time stepping [25]. Thus, the past research chose the single particle’s ball diameter (Figure 1a) and did not choose one of the joint particles (Figure 1b) to calculate the particle gradation.

This past simulation has two problems: First, a circular particle (Figure 1a) is not equivalent to a soil particle (Figure 1b). The joint particles are better than the circular particles in representing the soils/rock (Figure 1d). Second, a circular particle’s particle size (Figure 1a) and that of a joint particle (Figure 1b) are different. The particle diameter (Figure 1a) cannot be used for gradation calculation because actual soil particles are joint particles (Figure 1b); they are not a circle. The joint particles, not a single circular particle, go through the sieve hole during the soil particles’ screening experiment. The joint particle size, not the circular particle size, should be chosen to calculate the gradation. The joint particle (Figure 1b) is the actual particle of soils/rock. This paper aims to solve the above problems, proposes a discrete element, i.e., a joint particle model for soil simulation, and develops a joint particle size calculation method. We used this model and calculation method to study the soil porosity at some depths. Obtain the characteristics of the elastic modulus, porosity, and Poisson’s ratio with the depth. The circular discrete element is presented in this research and designed to simulate a soil material more realistically.

## 2. Joint Particle Model and Joint Particle Size

### 2.1. Joint Model for Soils

We used many joint soil particles to simulate soil. The paper aims to realize that joint soil particles are cemented and bonded by circular particles to form soil. The number of circles creating a joint soil particle is not the focus of this paper, although scientists need further analysis at this scale. The number was 3–6 in this research. These ball particles connect. One ball particle needs to connect at least one of the other particles. If a ball particle does not relate to any other ball particles, it is dangling and hanging, and it does not belong to this joint particle.

**Figure 2:**
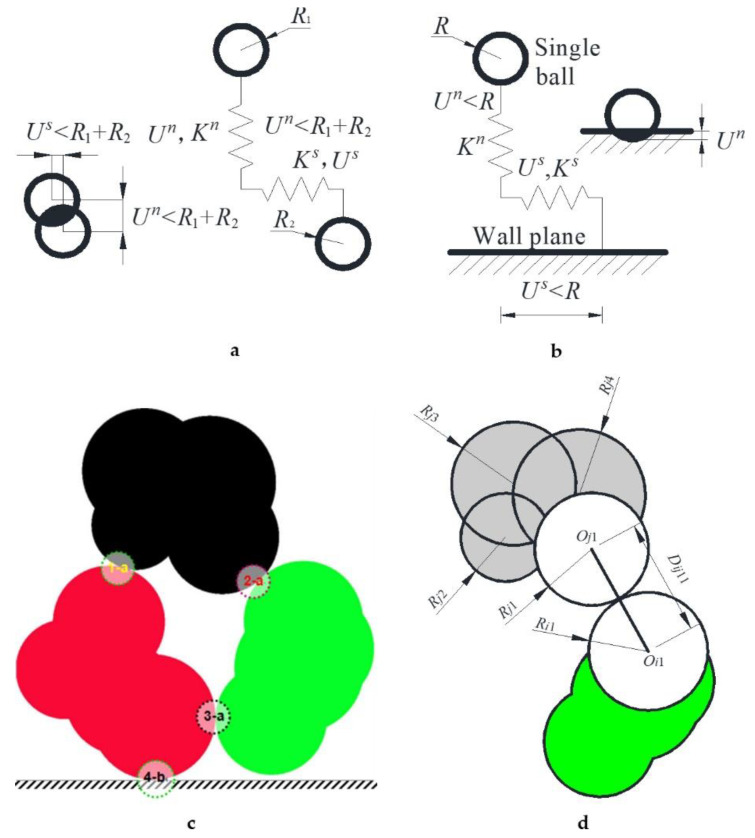
Mechanical relationship between ball particles and walls (**a**). mechanical model of interaction between single ball particles; (**b**) mechanical model of interaction between particle and wall plane. (**c**) the relationships. *R*_1_, *R*_2_ are the radii of single particles; *K^n^* is the elastic stiffness, and *K^s^* is the shear stiffness. The black shaded area overlaps the ball particles between the ball particles and the wall plane. Three joint particles are red, green, and black, which contact each other. There is a wall plane at the bottom. **1-a, 2-a** and **3-a** are three contact points between two particles: the contact relationship in (**a**). **4-b** is the contact point between particle and wall, the contact relationship in (**b**); (**d**) determination of distance, contact, and extrusion of joint particles.

In the numerical simulation test of soil mechanics, we restricted the two sides of the rectangular soil by a wall, and applied pressure through the wall in the soil’s upper part. The mechanical interaction between the soil particles (Figure 2a) and between the particles and the wall plane (Figure 2b,c) involves two effects: pressing and shear sliding translation, simplified into the mechanical model shown in Figure 2. Figure 2d offers the connection and contact between balls connected with two joint particles.

Pressing between two single ball particles means that the distance between the two balls is less than the sum of their radii (Figure 2a). When the distance is greater than or equal to the two radii’s sum, the two balls’ mechanical action disappears. The interaction between a single circular particle and the wall plane is similar (Figure 2b). The mechanical relationship is *F^n^* = *K^n^U^n^*, *F^s^* = *K^s^U^s^*, where *F^n^* and *F^s^* are the normal force and tangential force between two particles or between a particle and a wall plane, respectively. *U^n^* is the compression displacement between particles or between a particle and the wall plane. *K^n^* is the elastic stiffness. *U^s^* is the sliding shear displacement between particles or a single particle and a wall plane. *K^s^* is the shear stiffness of this shear.

*O_j_*_1_, *O_j_*_2_, *O_j_*_3,_ and *O_j_*_4_ are the 1st, 2nd, 3rd, and 4th circular particles in the *j*th joint particle (Figure 2d). *O_i_*_1_ is the first circular particle in the *i*th joint particle. The relationship between the distance of two circles’ centers and the sum of the two radii is needed to judge whether two joint particles are in contact; to calculate the coincidence degree of mutual extrusion, *R_im_* is the mth circular particle radius composed of the *i*th joint particle (as shown in Figure 2d). *R_jn_* is the radius of the *n*th circular particle composed of the *j*th joint particle. *D* is *U* in Figure 2a. When *D_ijmn_* > *R_i_*_m_ + *R_jn_*, two joint particles do not contact each other. When *D_ijmn_* = *R_i_*_m_ + *R_jn_*, two joint particles just contact. When *D_ijmn_* < *R_i_*_m_ + *R_jn_*, two joint particles squeeze against each other. *m* = 1, 2, …, *n* = 1, 2, …, the maximum value of *m* and *n* is the number of circular particles in the joint particle of the *i*th and *j*th joint particle.

### 2.2. Joint Particle Size: Rotation Calculation Model

**Figure 3:**
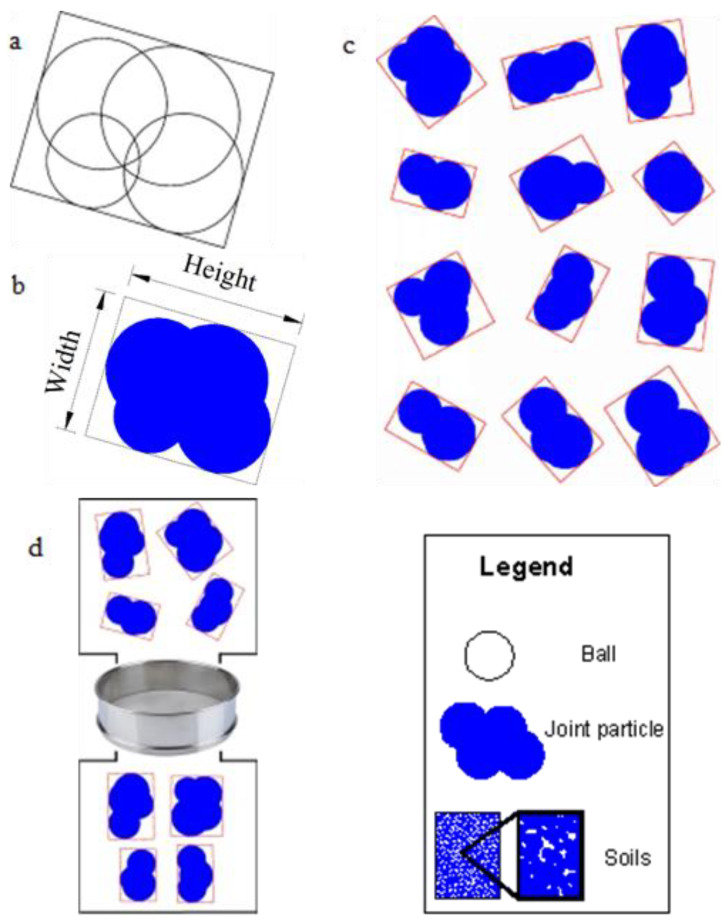
Joint particle size and screening process. (**a**) Four ball particles composite a joint particle; (**b**) the maximum width rectangle covering the joint particle; (**c**) some joint particles with its own the maximum width rectangle; (**d**) joint particles passing through the sieve by rotation at the maximum width of its rectangle; the solid ball is the elementary ball; the solid black block is the soil particle. Soils consist of many joint soil particles; joint soil particles consist of solid balls. Width and height denote the joint soil particle’s width and height, respectively, where Height ≥ Width.

Some circular balls (Figure 3a) are connected to form a joint soil particle (Figure 3b). Some joint particles cluster into soils, which have a specific porosity. Therefore, the circular ball is the elementary unit. These balls, making up a joint particle, have a high connection strength with the other balls in the same soil particle. The stability between balls is high enough to prevent their connections from being broken when soil is under stress to a great extent. More types of joint soil particles are in Figure 3c.

The soil comprises many joint soil particles (Figure 1b), mainly measured and evaluated by the gradation curve. The cumulative percent by the weight of soil passing through a given sieve is the percent of finer. The literature [26,27] showed the gradation curve’s details. Choose the uniformity coefficient and the gradation coefficient according to the gradation curve. The uniformity coefficient is *C_u_ = D*_60_/*D*_10_; the gradation coefficient is *C_c_* = (*D*_30_)^2^/(*D*_60_*D*_10_), where *D*_60_, *D*_30,_ and *D*_10_ are the diameters through which 60%, 30%, and 10% of the total soil mass pass. One must obtain the particle-size distribution of the simulated soil particles constituting the soil to determine the parameters of *D*_60_, *D*_30_, *D*_10_, *C_u_* and *C_c_*.

The joint particle size is not the size of the balls (the ball diameter); instead, the particle size is the minimum sieve size that passes through. Furthermore, the soil particles’ particle size distribution is determined by a sieving test, in which soil particles are passed through a standard sieve, as shown in Figure 3d. The particles passing through the mesh are smaller than the sieve’s pore diameter. As a result, the particles remaining on the sieve have larger diameters than the pore diameter of the sieve.

In calculating the particle sizes of joint particles, we considered many rectangles to cover the joint particles. These rectangles cover an entire joint particle and were tangent to the particle boundary. The rectangle with the smallest width (rectangle in Figure 3a–d) among all the rectangles covering the particles was determined. Next, we sieved the joint particles according to Figure 3d; the particles could pass through the sieve only vertically according to the minimum width. If the minimum width of the rectangle was precisely equal to the sieve’s diameter, the soil particles could pass through the sieve, and the minimum width is the particle size of the joint soil.

**Figure 4:**
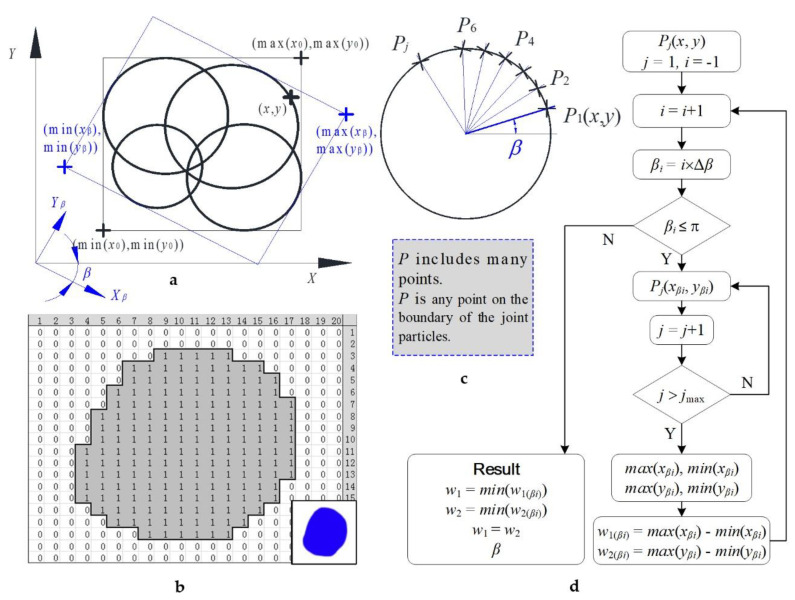
Rotation calculation model of the coordinate system to determine the soil particle size. (**a**) The calculation model for digital joint soil particles composed of balls; (**b**) the method for calculating the particle size of pixel joint particles of soil; (**c**) the points on the boundary of one joint particle; (**d**) programming flowchart.

This research proposes a rotation calculation model, as shown in Figure 4. We used the model in two types of conditions. The first condition involved joint soil particles composed of circles, as shown in Figure 4a. The points on the ball boundary had explicit numerical coordinates. Therefore, we only chose boundary points for calculation. The second condition concerned the pixel joint soil particle, as shown in Figure 4b. The dots on the soil particles are pixels, and the joint particle is the picture with pixels. In this method, we chose boundary points and we chose inside points for calculation.

Regarding the joint soil particles composed of circles, the joint particle size is determined by a rotating coordinate system, as shown in Figure 4a. The point coordinates (*x*, *y*) on the ball particles were determined, after which we found the new coordinate point (*x_β_*, *y_β_*) after the coordinate system rotated an angle *β* (Figure 4c,d). The relationship between the coordinates of the two coordinate systems is as follows: *x_β_ = x ×* cos*β − y* × sin*β*, *y_β_ = x* × sin*β + y* × cos*β*, where *β*∈ [0, π].

For a given *β* in Figure 4c,d, we found the max(*x_β_*), min(*x_β_*), max(*y_β_*) and min(*y_β_*) of the coordinates of all points on the boundaries of all circles, where the two side lengths of rectangles were *w*_1*β*_ = max(*x_β_*) − min(*x_β_*) and *w*_2*β*_ = max(*y_β_*) − min(*y_β_*). When *β*∈(0, π), min(*w*_1*β*_) and min(*w*_2*β*_) can be obtained. These two values should be equal, i.e., min(*w*_1*β*_) = min(*w*_2*β*_) = *w*. Here, *w* is the minimum width of the joint soil particles and reflects the joint soil particles’ particle size. *P*(*x*, *y*) includes many points. *P*(*x*, *y*) is any point on the joint particles’ boundary as shown in Figure 4c.

Similarly, if there are blue particles with arbitrary shapes, as shown in Figure 4b, they comprise blue pixels and other white pixels set to either blue RGB (0, 0, 255) or white RGB (255, 255, 255). We produced any color by blending and adding red, green, and blue (RGB). For example, RGB (200, 225, 255) means that the red color is 200, the green color is 225, and the blue color is 255. The blue color of the two colors, RGB (0, 0, 255) or white RGB (255, 255, 255), has the same number, 255. The first number of RGB, the red color position, was chosen for calculation. Of course, we chose the second number of RGB. The first RGB of the blue pixel position is 0, while the first RGB of the white pixel position is 255. We only changed the 255 into 1. Finally, we made Figure 4b. All points have a value of 1 inside the soils, and each point has corresponding coordinates. According to the calculation method shown in Figure 4b, after rotating the coordinate system by an angle *β*, calculate the widths *w*_1*β*_ and *w*_2*β*_ corresponding to the minimum rectangle covering a soil particle. When *β*∈(0, π), min(*w*_1*β*_) and min(*w*_2*β*_) can be obtained, the values of which should be equal, i.e., min(*w*_1*β*_) = min(*w*_2*β*_) = *w*, which is the particle size of the pixel soil particle. According to the image scale, obtain the joint soil particles’ actual particle sizes.

The programming flowchart is shown in Figure 4d. The result *w*_1_/*w*_2_ and *β*. Here, *w*_1_ and *w*_2_ are the minimum width of the joint soil particles and are less than or equal to the sieve diameter in Figure 3d. *β = β_i_* is the rotation angle from the initial position when *w* = *w*_1(*βi*)_.

Therefore, the particle size calculation in this paper is a process of discovering the minimum width rectangle. The simulation and indoor screening tests are well unified, establishing a foundation for simulating more realistic soil.

### 2.3. Porosity Estimation of Overlapping Particles: Pixel Counting Method

**Figure 5:**
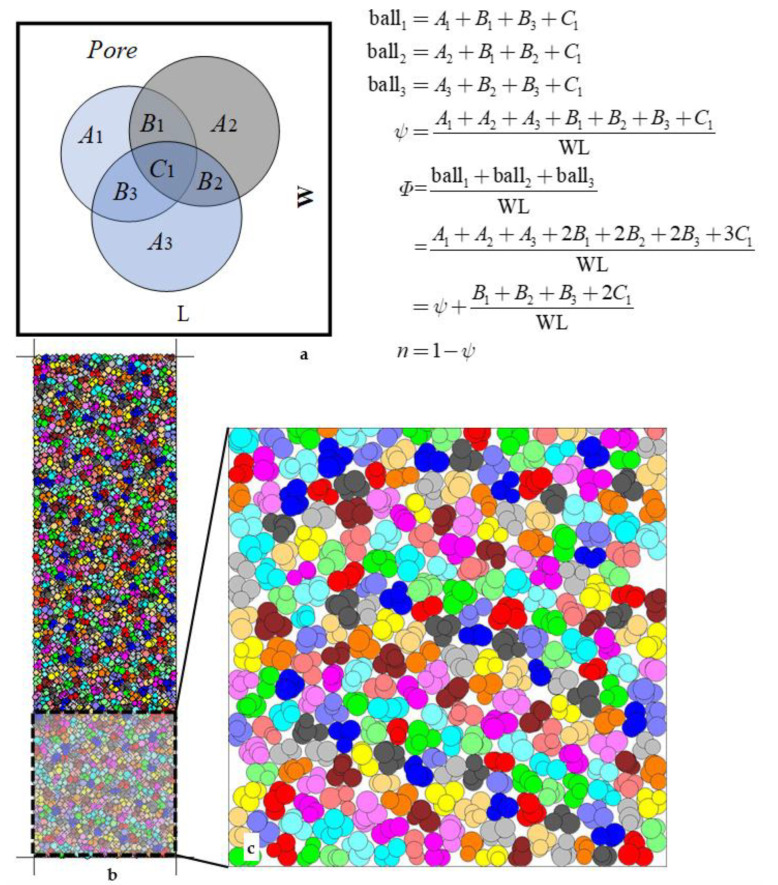
The joint soil particles form the porosity of the soil. (**a**) The overlap and relationship between balls compose one example joint particle; *A*, *B* and *C* are the areas of balls. ball*_i_*is *i* st ball area. W and L are the width and length of the example. *ψ* and *Φ* are the rate of the projected and the total area to the example area. *n* is the porosity. (**b**) compression diagram; **c** partial enlargement, with *n* = 0.1623.

The porosity of rock and soil in hydrogeology is an important physical parameter that affects and determines the permeability coefficient [28,29]. The porosity should be first calculated to obtain the relationship between soil porosity and pressure. There is no good way to get the porosity of discrete particles in past research [25,30,31]; more researchers choose to avoid or ignore the issue and to not determine the porosity. There was some overlapping areas (the shaded region shown in Figure 2b; B1, B2, B3, and C1 shown in Figure 5a). The porosity is the projected area’s rate, not the sum area of all balls in the example area. The porosity is *n* and 1-*ψ*, not 1-*Φ,* shown in Figure 5a. The fundamental difficulty is that the overlapping areas are hard to evaluate and calculate.

This research proposes a more accurate method (as shown in Figure 5) for calculating the soil particle area/volume to solve this problem: The pixel counting method. The soil is formed by accumulating soil particles by gravity (Figure 5b). The soil particles are colored (Figure 5c), while the background is white. That is, the soil pores are white. The white background RGB is (255, 255, 255). The RGB of colored particles is (R, G, B), where R ≠ 255 or G ≠ 255 or B ≠ 255. When circular particles appear in the overlaying case (Figure 2); they are still very colorful with the RGB (R, G, B). The pixel coordinates of the entire picture are PI(*x*, *y*). The *x* and *y* values are positive integers, where *x*∈ (*x*_min_, *x*_max_,) *y*∈ (*y*_min_, *y*_max_), *x*_min_, *y*_min,_ and *x*_max_, *y*_max_ are the minimum and maximum values of the *x*, *y* position coordinates in the picture. The total number of points of the color pixels is *S_B_* = ΣPI∣_(R__≠ 255 or G_ _≠ 255 or B__≠ 255)_, and the total number of points of the white pixel is *S*_W_ = ΣPI∣_(R = 255, G = 255, B = 255)_. The total number of pixels in the entire image is ΣPI = (*x*_max_ − *x*_min_) × (*y*_max_ − *y*_min_), where ΣPI = *S_B_* + *S_W_*; thus, the percentage of pores in the whole soil is *n* = *S*_W_/ΣPI, where *n* is the porosity of the soil after being compressed by an external force. The porosity in Figure 5 is 0.1623 after calculation.

### 2.4. Elastic Modulus and Poisson’s Ratio

We applied pressure to the soils in two directions at specific burial depths. According to the reference [12], we obtained the elastic modulus and Poisson’s ratio. We applied Fx and Fy forces in both directions; the stress is given by *σ_x_* and *σ_y_*, respectively. *Fx* and *Fy* are the force applied to the sample in *x* and *y* directions. *σ_x_* and *σ_y_* are the stress on the sample in *x-* and *y*-direction. The strain formulas are *ε_x_* = (*σ_x_* − *υσ_y_*)/*E* and *ε_y_* = (*σ_y_* − *υσ_x_*)/*E*. Because the problem is a plane strain problem, *ε_x_* = 0 and *σ_x_* = *υσ_y_*. Then, *σ_x_* + Δ*σ_x_* = *υ*(*σ_y_* + Δ*σ_y_*), and, thus, Δ*σ_x_* = *υ*Δ*σ_y_*. *ε_x_* and *ε_y_* are the strain on the sample in *x-* and *y*-direction. *υ* is Poisson’s ratio. *E* is the elastic modulus.

We applied a slight increase in the force Δ*σ_y_* to the soil sample when soils reached stability. Δ*σ_x_* can be obtained after the soils reach stability again under Δ*σ_y_*. In this way, *υ* can be obtained from Δ*σ_x_* = *υ*Δ*σ_y_*. Every pair of Δ*σ_y_* and Δ*σ_y_* values can correspond to a new value *ε_y_* + Δ*ε_y_*. We obtained Δ*ε_y_* from the soil deformation. Thus, we obtained the elastic modulus *E* according to Δ*ε_y_* = (Δ*σ_y_* − *υ*Δ*σ_x_*)/*E.*

## 3. Example

### 3.1. Joint Particle Size

**Figure 6:**
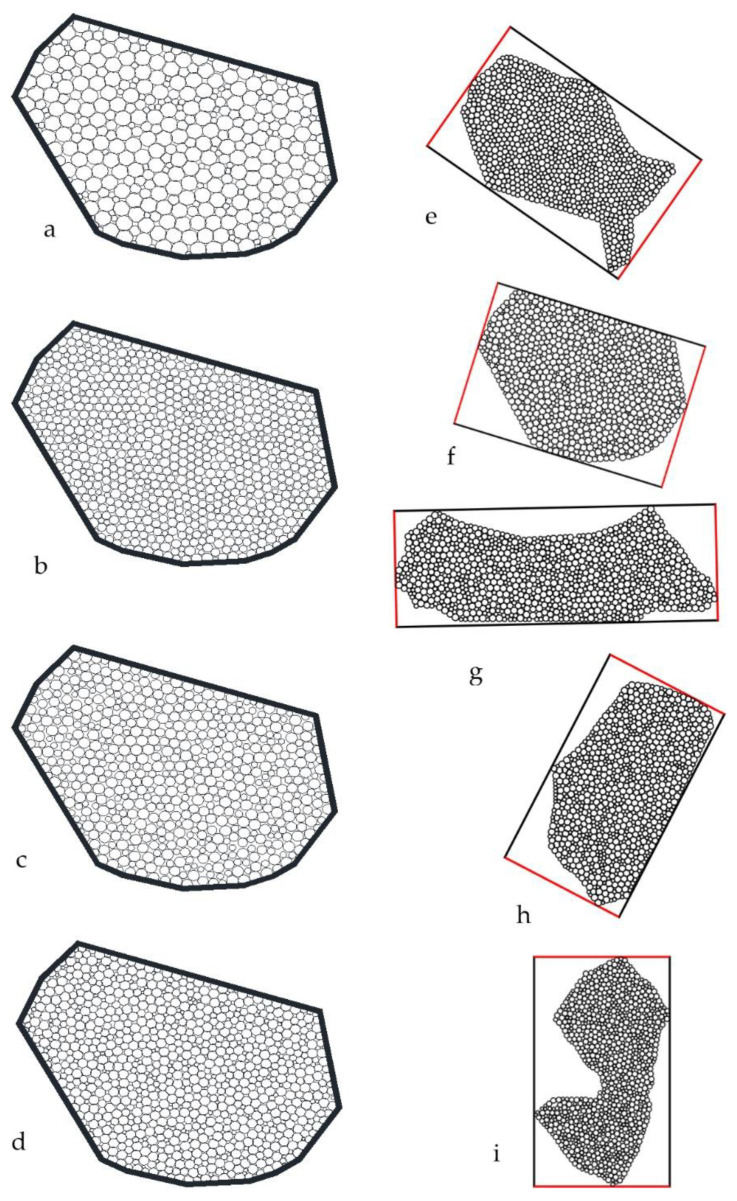
Different numbers of circular particles compose a joint particle and joint particle sizes (**a**–**d**) are the crystals 1 in Figure 1d generated by 400, 600, 800, and 1000 circular particles, respectively. (**e**–**i**) are the crystals 2, 1, 5, 3, and 4 in Figure 1 and are composed of 1000 particles. The width, height and counterclockwise rotation angle of the minimum width rectangle are, respectively (5.216, 8.346, 124.905), (3.707, 5.441, 107.143), (1.712, 4.737, 89.381), (2.499, 4.429, 26.929) and (2.337, 4.535, 11.4590).

According to the calculation method in the paper, the crystal particle in Figure 1 is simulated in Figure 6. As the number of circular particles increases in the joint particle, the joint particle shape can better fit the shape of the simulated particles. The crystals of 1, 2, 3, 4, and 5 in Figure 1 can be composed of many circular particles; these circular particles overlap and bond with each other to some extent. As shown in Figure 6, we calculated the particle size according to the method in Section 2.2. From the results, the way presented in this paper is very robust.

**Figure 7:**
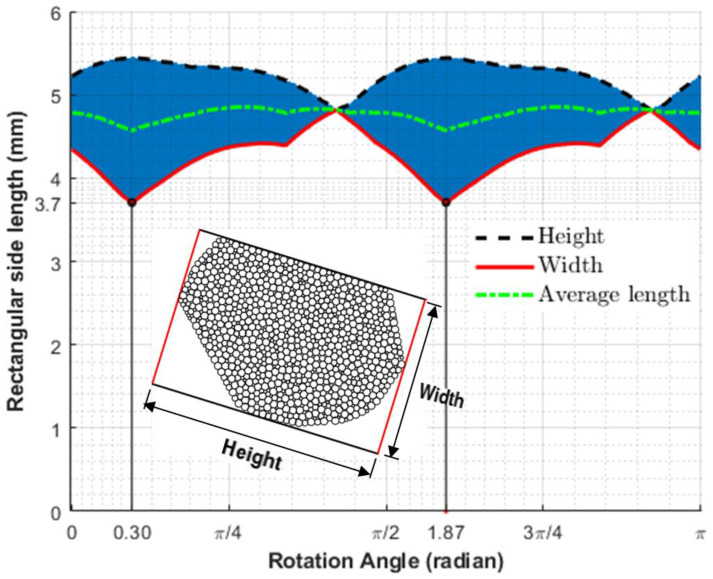
Particle size calculation of joint particles under different rotation angles. The graph of the rotation angle and the height/width of the corresponding rectangle with minimum width. The average length is (Height + Width)/2.

Choose crystal 1 in Figure 1 to calculate the particle size. Then, rotate the particle by an angle to find the length of the two rectangular sides. The long side is the height, and the short side is the width. According to the rotation angle and the two lengths, draw Figure 7.

In this paper, the particle size is the width of the minimum width rectangle, the minimum value of the solid red curve in Figure 7. The rectangle with the minimum width is (5.441 mm, 3.707 mm). The rotation angles are 0.30 and 1.87, that is, 17.189° and 107.143° with an error of 0.046°, which is acceptable. According to previous studies, the average size of the rectangle length is the dotted line. The minimum value is 4.574 mm. However, the green dotted line and the solid red line are different, and the two curves do not coincide. Therefore, the minimum values are 3.707 mm and 4.574 mm, which are not equal. According to Figure 3 and Section 2.2, the particle size in this paper is scientific and reasonable; the calculation process conformed to the test environment and conditions. Therefore, it is inappropriate to take the average value of the sum of the length of the previous value as the particle size. 

The length and width of the rectangle changed simultaneously with the rotation angle, as shown in Figure 7. It shows the rule of periodic change with period π/2. In the calculation, taking the value (0, π/2), not (0, π), to calculate the rotation range can meet the requirements. More joint particle lengths and widths have been calculated and a database built. Refer to the literature [32].

### 3.2. Particle Gradation and Porosity under Pressure

The weight of the upper soil compresses the soil at a specific depth. The circular particles in contact with each other once again overlap. Figure 2 shows the shadow region, and the pore sizes become small. We established the compressed result image and obtained the porosity according to the image pixel count method presented in Section 2.3.

Over a long geological history, the soil has gradually accumulated small particles. As a result, the thickness and upper pressure have steadily increased. The upper pressure imposed on a specific soil depth is related to gravity’s effect on the upper soil. *P* = *ρgh*, where *ρ* = *ρ*_s_(1 − *n*), *P* is the upper pressure, *ρ* is the soil density, *ρ*_s_ is the soil particle density, *g* is the gravitational acceleration, *h* is the depth, and *n* is the soil porosity. Furthermore, we obtained the relationship between the upper pressure and porosity.

We chose many physical parameters (Figure 2) [33,34]. The ball density *ρ* is 2300 kg/m^3^. The normal contact stiffness *K^n^* is 4.4 × 10^7^ N/m, and the shear contact stiffness *K^s^* is 2.2 × 10^7^ N/m. The ratio of the two stiffness *K^s^/K^n^* is 0.5. The gravitational acceleration *g* is 9.81 m/s^2^, the ball radius *r* is 2–3 mm, and the friction coefficient *Fr* is 0.5.

**Figure 8:**
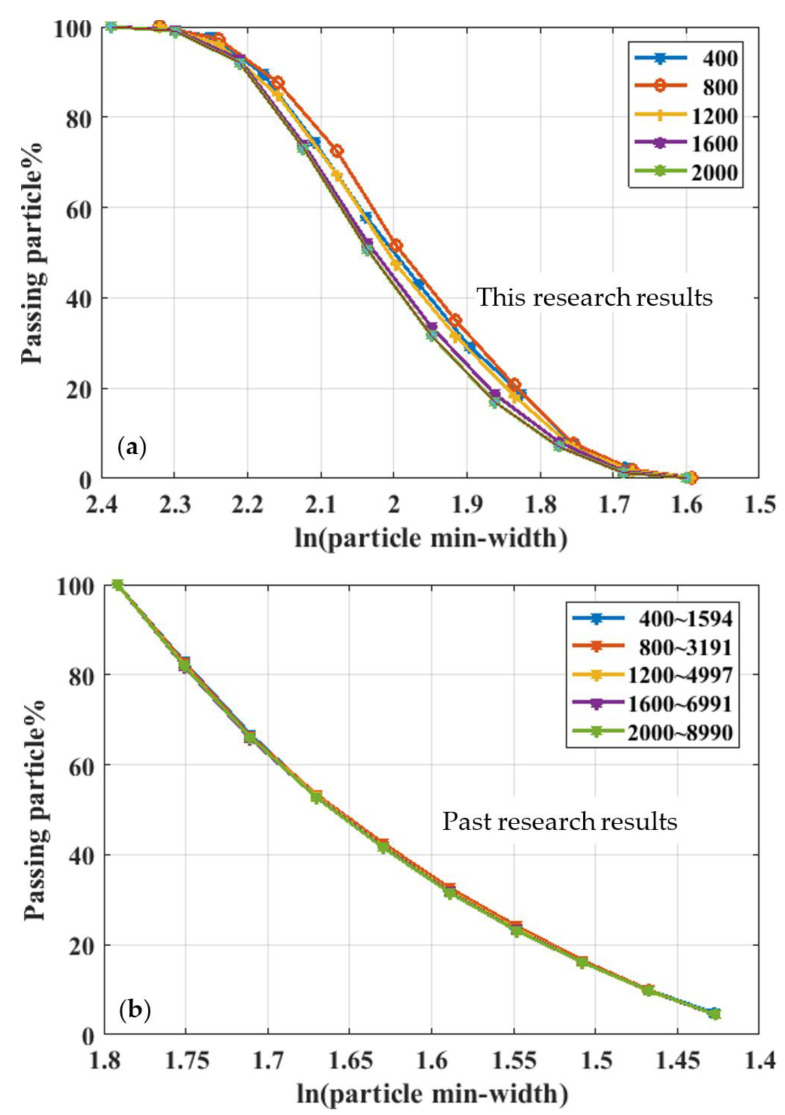
The uniformity coefficient, gradation coefficient, and gradation curve of soils. The particle min-width unit is mm. (**a**) Combined particle soil gradation curves calculated by the rotation calculation model; (**b**) single-ball soil gradation curves, where 400, 800, 1200, 1600, and 2000 denote the number of combined particles and single balls. For example, 400~1594 indicates that 1594 single balls constitute the 400 combined particles. The min-width unit is mm. The *X* coordinate is ln(min-width).

This paper presents an example calculation. Because the calculation needs to consume a lot of calculation time, we chose the joint particles composed of 3–5 circular particles for research in this paper. We simulated other, more circular, particles in the same method and steps. The research process was as follows: (1) Generate two thousand joint particles (Figure 1b and Figure 3b,c). The two thousand joint particles comprise circular particles (Figure 1a and balls in Figure 3) sized and located randomly according to a specific distribution method, such as the normal, index, and lognormal, uniform distribution. We generated the circle center by the normal distribution in the following example, and the uniform distribution chose the radii. (2) A certain number of soil particles (Figure 1b and Figure 3b,c) were randomly selected from the 2000 soil particles and were allowed to accumulate under the effect of gravity. (3) We applied the pressure to the upper part of the generated soil. The magnitude of the pressure corresponded to the effect of gravity on the soil in this depth range. (4) We calculated the soil porosity under this pressure. We obtained the relationship between the upper pressure and the porosity. In the range of *x*∈(−0.02 m, 0.1 m), *y*∈(−0.02 m, 1.5 m), 400, 800, 1200, 1600, and 2000 soil particles were selected, and *C_u_* and *C_c_* were calculated according to Section 2.2. The results are listed in Table 1 and shown in Figure 8.

The particle min-width in Figure 8a is the joint particle (Figure 3 and Figure 4) size of soils, the minimum rectangle’s minimum width covering the joint soil particle. The particle min-width in Figure 8b is the single ball’s radius (Figure 3 and Figure 4).

*C_u_* and *C_c_* in Table 1 of soils were made up of two types of particles. *D*_60_, *D*_30,_ and *D*_10_ were the sieve diameter, with 60%, 30%, and 10% of the total soil mass passing through the sieve (unit: mm). *C_u_ = D*_60_/*D*_10_ was the uniformity coefficient. *C_c_* = (*D*_30_)^2^/(*D*_60_*D*_10_) was the gradation coefficient. *C_cc_* and *C_cs_* were *C_c_* of the combined particles and the single ball, respectively. *C_uc_* and *C_us_* are the *C_u_* of the combined particles and the single ball, respectively. | | denotes the absolute value. *C_u_ = D*_60_/*D*_10_ is the uniformity coefficient. *C_c_* = (*D*_30_)^2^/(*D*_60_*D*_10_) is the gradation coefficient. *C_u_*, *C_c_* are important parameters in particle, soil, and sand research, and engineering [35]. We used the two parameters to distinguish between well graded and poorly graded coarse-grained soil using laboratory tests of the grain size distribution [36]. The two parameters satisfy the requirements in engineering projects or science research. We can say that the materials were qualified [37].

Figure 8a is a gradation curve for the joint particles, similar to some of the literature’s gradation curves [38,39]. Figure 8a and Table 1 show that the five gradation curves were not different, and that the difference between *C_u_* and *C_c_* was not significant. Therefore, the gradation curve was not closely related to the number of selected soil particles. Figure 8b is a gradation curve for ball particles and not joint particles. Figure 8b and Table 1 show that the five gradation curves were almost coincident. Figure 8a,b shows that the particle size in Figure 8a was larger than the particle size in Figure 8b and that the corresponding gradation curves were significantly different. Therefore, we did not take the ball particle gradation curve as the joint soil particles’ gradation curve, which is different from previous research methods [12].

**Figure 9:**
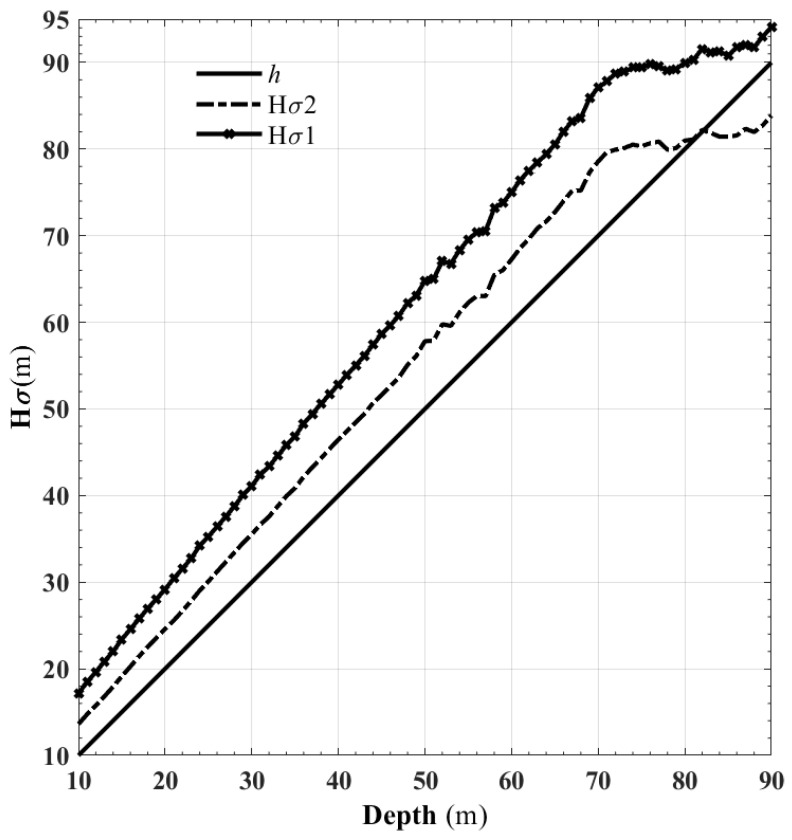
Equivalent depth of upper and lateral pressure at different depths and increments.

**Figure 10:**
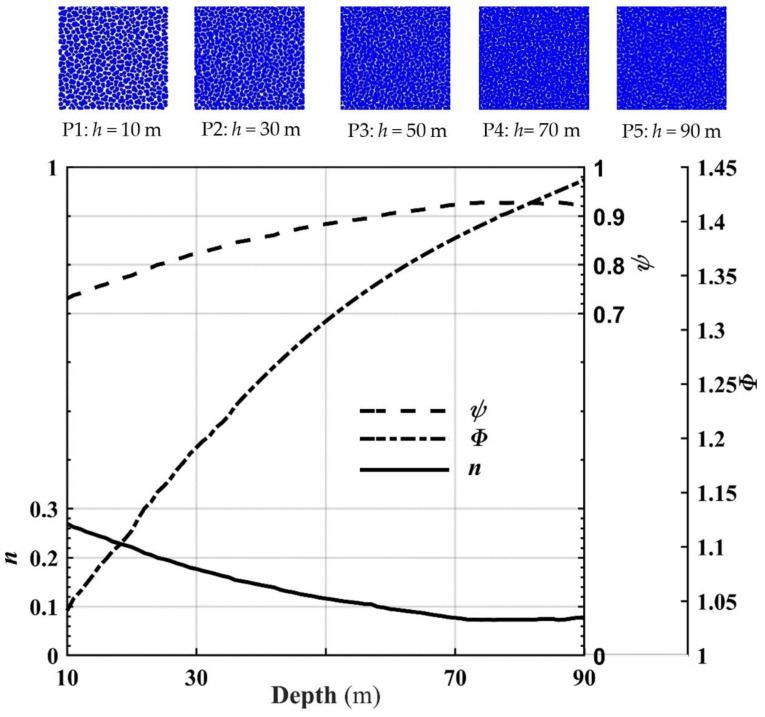
The soil porosity curve for different depths P1, P2, P3, P4, and P5 are soil particles’ morphologies under pressure due to the upper soil weight. The blue areas represent the soil particles, while the white areas depict the pores between the particles. P1: *h* = 10 m, *n* = 0.2694; P2: *h* = 30 m, *n* = 0.1772; P3: *h* = 50 m, *n* = 0.1165; P4: *h* = 70 m, *n* = 0.0769; and P5: *h* = 90 m, *n* = 0.0763.

**Figure 11:**
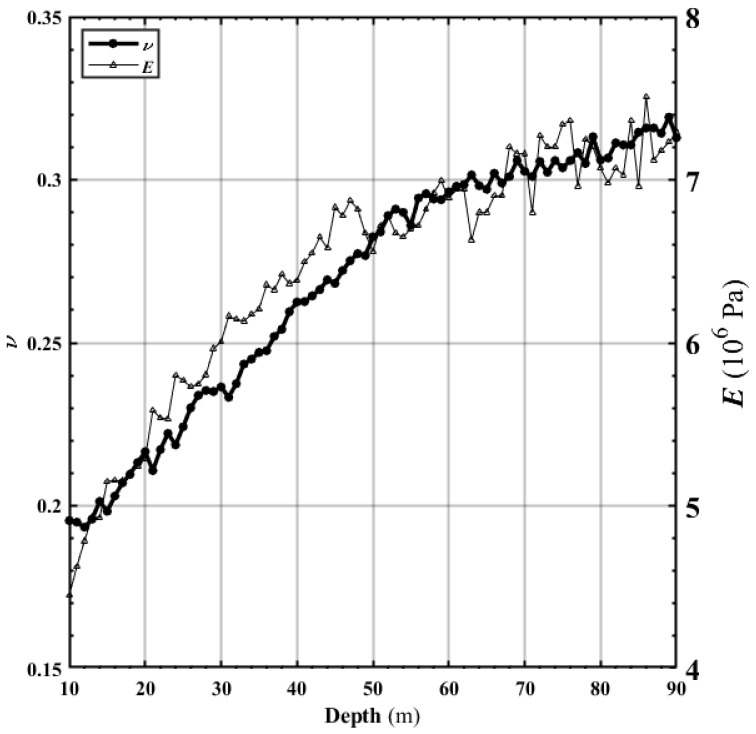
Poisson’s ratio and elastic modulus of soil at different depths.

This gradation curve of joint particles in Figure 8a has not been calculated and displayed in previous research. The reason is that the particle size of joint soil particles could not be well estimated. We obtained the particle size of the soil particles via the calculation method of this paper. First, we determined the gradation curve of the granular soil. After compression, the range of images taken was *x*∈(−0.02 m, 0.1 m), y∈(−0.02 m, 0.1 m), and the depth ranged from 10 m to 90 m, with an interval of 1 m. We recorded the force on the upper, lower, left, and proper boundaries and the sample’s height with the constant increase in the upper pressure. The compressed soil picture calculated the porosity using the pixel recognition technology discussed in Section 2.3. Figure 9 and Figure 10 show the boundary pressure and porosity. Next, we obtained the elastic modulus and Poisson’s ratio according to the calculation procedure mentioned in Section 2.4, as shown in Figure 11.

We fit the following formula to the porosity and depth in Figure 10.

(2) $$n=a\cdot b^{Depth}+c$$

*a* = 0.311, *b* = 0.973, *c* = 0.0378; the correlation coefficient *R* was 0.998.

We pressurize the sample from the top and restricted the displacement on both sides. The pressures above and on both sides were *σ*_1_ and *σ*_2_, respectively. *H_σ_*_1_ and *H_σ_*_2_ are the pressure conversion depths of *σ*_1_ and *σ*_2_, respectively, where *H_σ_*_1_ = *σ*_1_/(*ρg*) and *H_σ_*_2_ = *σ*_2_/(*ρg*); *h* is the depth; *ρ* is the soil density; *g* is the acceleration due to gravity.

Figure 9 shows that the change law of the upper and lower pressures had a good consistency. *H_σ_*_1_ was more significant than *H_σ_*_2_, and both forces were greater than the natural depth *h*. The slope of Line *h* is 1. The slopes of Line *H_σ_*_2_ and Line *H_σ_*_1_ gradually increase with the depth increasing. After 70 m, the slopes of Line *H_σ_*_2_ and Line *H_σ_*_1_ tend to decrease, at approximately 80 m, Line *H_σ_*_2_ begins to fall below the *h* line.

Curve *n* and curve *ψ* in Figure 8 are results obtained by this research, while curve *Φ* is the result ignored in past research. The directional area accumulation of balls is not the projected area; the porosity *n* was 1-*ψ*, not 1-*Φ* from Figure 5a and Figure 10.

The curve *n* in Figure 10 shows that the porosity gradually decreased as the depth increased. The porosity gradually reduced with the depth increasing from the image analysis of the soil in P1, P2, P3, P4, and P5. Figure 10 shows the following: (1) when the depth was small, some interconnected pores existed between the soil particles, and the porosity was large; (2) when the depth was significant, the particles were superimposed on each other, the connected pores decreased in number or even disappeared, the pore sizes gradually decreased, and the porosity decreased. This characteristic is consistent with the literature’s conclusion that the density increases [40,41,42,43]. Thus, the density of soil after compression increased, while the porosity decreased. From a depth of approximately 72 m, the porosity curve tended to be stable; from about 80 m, the porosity tendd to increase slightly. The soil particles’ elastic modulus and Poisson’s ratio gradually increased as the depth increased (Figure 11). The elastic modulus and Poisson’s ratio underwent some upward and downward fluctuations. At a depth of about 48 m, the elastic modulus had more fluctuations.

In contrast, Poisson’s ratio maintained a relatively steady increase. The fluctuation of the elastic modulus was more significant than that of Poisson’s ratio. With the depth increase, Poisson’s ratio fluctuated when about 62 m.

## 4. Discussion

As the soil depth increased, the upper pressure increased, the distance between the soil particles gradually decreased, many soil particles appear to overlap (from the progression order in P1, P2, P3, P4, and P5 in Figure 10), and the pore sizes between the soil particles decreased. Therefore, as the depth of burial increased, the porosity (shown in Figure 10) gradually decreased.

With more overlap between soil particles, the soil’s ability to resist external force deformation was enhanced. Under the same external pressure and stress *σ*, the deformation and strain *ε* became smaller. According to the formula *E* = *σ*/*ε*, the elastic modulus (Figure 11) gradually increased as the depth increased. The parameters in Equation (2) can preliminarily clarify the physical meaning: Parameter *c* is the porosity when the Depth → ∞, which indicates that no pores exist between the particles, the porosity only pertains to the pores inside the material, and this part is related to the microscopic morphology inside the material. When depth → 0, *a* + *c* is the porosity; the soil porosity is *n_0_* in an entirely pressure-free and loose state. At that time, *a* = *n*_0_ − *c* is related to the loose state of the material. *B* represents the degree of porosity increase with the depth increasing. *B* has a direct relationship with the elastic modulus *E* of the material and the density *ρ* of the particles and is, thus, related to the material’s mechanical parameters.

At 70 m, the porosity tended to be stable. From 80 m, the porosity curve increased slightly. The fluctuation occurred because the particles detached from the soil [10]. In calculating the PDEM, we applied the external force to the soil particles by moving the wall toward the soil [44]. As the wall moved toward the soil, it compressed the soil. As a result, the interaction force between the soil particles and the wall gradually increases. According to Figure 2b and the formula *F^n^* = *K^n^U^n^*, *U^n^* must be less than *R.* As the pressure increased, *K^n^* remained stable during the calculation; *U^n^* gradually increased. When *U^n^* > *R*, the black shaded area in Figure 2b exceeded half the circle; the circle center passed through the wall. The force between the ball and the wall changed to *F^n^* = *K^n^*(2*R* − *U^n^*), and the direction changed by 180°. The ball immediately passed through and away from the wall.

The elastic modulus and Poisson’s ratio volatility in Figure 11 are greater than the porosity volatility in Figure 10. Under the above pressure, the particles are superimposed and elastically deformed. When the particles escape, the force on the boundary changes immediately. According to the formula Δ*σ_x_* = *υ*Δ*σ_y_* and Δ*ε_y_* = (Δ*σ_y_* − *υ*Δ*σ_x_*)/*E* [12], the elastic modulus and Poisson’s ratio change drastically. However, space opened up by particles’ ejection is immediately occupied by the elastic release and deformation recovery of other elastically deformed particles. As a result, the superimposed elastic area between the particles (as shown in Figure 2a,b) was diminished. The release of the particle superposed area makes up for the space left by escaping particles. The particle area in the soil does not change much; thus, the soil porosity does not change significantly.

The force in the *y* direction produced a strain *ε_y_* in *y* direction. Similarly, the force in the *x* direction produced a strain *ε_x_* in the *y* direction. The *y* direction was the primary stress direction. Poisson’s ratio is the ratio of the lateral strain *ε_x_* to the active strain *ε_y_*, i.e., *υ* = *ε_x_*/*ε_y_*. When the pressure in the active direction was slight, the specimen was not prone to the lateral strain; Poisson’s ratio was relatively small. However, as the pressure increased and the particles were pressed and superimposed, the entire soil became more compact. After the strain occurred in the active direction, the lateral direction was more prone to strain. As a result, Poisson’s ratio increased. Therefore, Poisson’s ratio increased with the burial depth and the upper load applied to the soils (as shown in Figure 11).

## 5. Conclusions

This paper was founded on the model and method, the rotation calculation model for the particle size and pixel counting method for the porosity. Further, we obtained the particle gradation of joint particles. The two models overcame past research shortcomings, e.g., considering soil particles as only circles and balls [45,46,47,48], ignoring the particle size and particle gradation of joint soil particles and the actual porosity. This research enables the development of more realistic discrete elements and helps to simulate the more complex rock and soil materials. In particular, this study provides a good reference for the transport of foundation sediment in porous media [49] and containing faults [29].

The founded calculation model combined with the discrete element force calculation. The relationship between porosity, elastic modulus, Poisson’s ratio, and soil pressure (the buried depth) was studied. Soil, composed of soil particles, was compressed by an external force. The compactness, Poisson’s ratio, and the elastic modulus increased, while the porosity decreased, consistent with previous research results [50,51].

When soil particles were ejected from the soil, and the depth was more than the sensitive depth values, Poisson’s ratio, the elastic modulus, and the porosity had significant fluctuations, but the elastic modulus changed significantly. Therefore, the elastic modulus is a good index and is the physical parameter for primary monitoring in the integrity monitoring of underground petroleum and groundwater reservoirs.

This research focused on the model, calculating the particle size of soil particles and studying the relationship between compression porosity, the elastic modulus, Poisson’s ratio, and depth. We did not consider the ball radius size and the number when simulating soil particles, and, therefore, did not consider the scale of the soil particle size. Simulating soil particles using different radii and different numbers of balls may yield different final results and be considered in subsequent studies.

## Figures and Tables

**Table 1:** *C_u_* and *C_c_* of soils made up of two types of particles.

Number of Particles	Combined Particles					Single Ball					Error	
	*D*_10_	*D*_30_	*D*_60_	*C_c_*	*C_u_*	*D*_10_	*D*_30_	*D*_60_	*C_c_*	*C_u_*	*|C_cc_-C_cs_|/C_cc_* *%*	*|C_uc_-C_us_|/C_uc_* *%*
400	5.87	6.692	7.744	0.985	1.319	4.342	4.842	5.427	0.995	1.250	1.016	5.245
800	5.881	6.605	7.605	0.975	1.293	4.340	4.840	5.430	0.994	1.251	1.951	3.243
1200	5.934	6.731	7.758	0.984	1.307	4.349	4.854	5.435	0.997	1.250	1.322	4.375
1600	6.009	6.885	7.904	0.998	1.315	4.344	4.864	5.441	1.001	1.252	0.301	4.766
2000	6.082	6.961	7.943	1.003	1.306	4.344	4.864	5.435	1.002	1.251	0.100	4.206
